# Evaluating mean platelet volume and platelet distribution width as predictors of early-onset pre-eclampsia: a prospective cohort study

**DOI:** 10.1186/s40748-024-00174-8

**Published:** 2024-03-01

**Authors:** Patience Ijeoma Udeh, Ayokunle Moses Olumodeji, Taiwo Olufunmilayo Kuye-Kuku, Oluwaseun Olubowale Orekoya, Olufemi Ayanbode, Adetokunbo Olusegun Fabamwo

**Affiliations:** 1 Department of Obstetrics and Gynaecology, Lagos State University Teaching Hospital, Lagos, Nigeria; 2 Department of Obstetrics and Gynaecology, Lagos State University College of Medicine, Lagos, Nigeria

**Keywords:** Early-onset pre-eclampsia, Platelets, Platelet indices, Pregnancy

## Abstract

**Background:**

Platelets are pivotal players in the pathophysiology of pre-eclampsia, with observed lower counts in affected individuals compared to normotensive counterparts. Despite advancements, the elusive cause of pre-eclampsia persists, motivating intense global efforts to identify reliable predictors. The currently recommended predictors of pre-eclampsia are not readily available in many resource-limited regions like Nigeria. This cohort study explores the potential of mean platelet volume (MPV) and platelet distribution width (PDW) as predictive markers of early-onset pre-eclampsia. Both platelet indices are components of the full blood count, a widely available routine test in pregnancy.

**Methods:**

In this prospective cohort study, 648 healthy pregnant women attending antenatal care at Lagos State University Teaching Hospital and General Hospital Ifako-Ijaiye, Lagos, were recruited between 14-18weeks gestational age. Platelet count (PC), MPV and PDW were measured from their venous blood at recruitment. Participants were monitored until 34weeks of gestation, focusing on the occurrence of early-onset preeclampsia as the outcome of interest. Individuals with chronic medical conditions were excluded from the study. Data analysis involved t-test, Chi-Square and Mann–Whitney U tests, with statistical significance set at a confidence level of 95% and *p* < 0.05. Sensitivity, specificity, and predictive values were determined using receiver operating characteristics (ROC) curves.

**Results:**

The incidence of early-onset pre-eclampsia in the study was 5.9%. Women who later developed pre-eclampsia had higher median MPV and PDW at 14-18weeks (10.8 fl. and 24.8 fl.) compared to normotensive women (8.1 fl. and 13.3 fl.)(*p* < 0.001). The median PC was lower in pre-eclamptics (190 × 10^3^/µl) compared to normotensives(264 × 10^3^/µl)(*p* < 0.001). Using Youden’s test, cut-off values identified: PC < 211.5 × 10^3^/µl, MPV > 9.4 fl., and PDW > 21.3 fl., predicted early-onset pre-eclampsia with 96.6% sensitivity and 65.6% specificity for PC; 79.3% sensitivity and 97.7% specificity for PDW; and 82.8% sensitivity and 96.1% specificity for MPV. Cut-offs of PC < 185 × 10^3^/µl, MPV > 10.7 fl., and PDW > 28.3 fl., predicted severe early-onset pre-eclampsia with 100.0% sensitivity and 90.9% specificity for PC, 100.0% sensitivity and 99.4% specificity for MPV, and 100.0% sensitivity and 99.8% specificity for PDW, with corresponding area under the ROC curves of 0.983, 0.996, and 0.998, respectively.

**Conclusion:**

The evaluation of MPV and PDW between 14 and 18 weeks of gestation appears to be a reliable predictor of severe early-onset pre-eclampsia.

## Introduction

Pre-eclampsia, characterized by hypertension and end-organ damage during pregnancy, remains a leading cause of maternal and perinatal morbidity and mortality globally [1]. Despite extensive research, the precise etiology of pre-eclampsia remains elusive, hindering the development of targeted prevention and management strategies [2]. Early-onset pre-eclampsia, distinct in its onset before 34 weeks of gestation, poses a more significant threat to both maternal and fetal well-being, emphasizing the critical need for accessible and effective predictive markers in regions with limited healthcare resources [3].

Preeclampsia is a multifactorial condition that negatively impacts several important organs in pregnant women. It increases the health risks for both the fetus and the pregnant woman, leading to more complications and potential harm [4]. Early-onset preeclampsia is particularly concerning as it significantly raises the risk of serious morbidity affecting the heart, lungs, central nervous system, kidneys, liver, and other organs [4]. In a study by Linoskova et al., they examined data from 670,120 deliveries over nine years to understand the patterns of early-onset versus late-onset preeclampsia and its connection to severe maternal health problems [3]. Their findings revealed higher rates of maternal deaths among women with early-onset preeclampsia (42.1 per 100,000 deliveries) and late-onset preeclampsia (11.2 per 100,000) compared to women without preeclampsia (4.2 per 100,000) [3].

A novel first-trimester screening algorithm, validated to predict preterm preeclampsia, incorporates mean arterial blood pressure, Doppler ultrasound-measured maternal uterine artery resistance, and Placental Growth Factor (PlGF) levels [5, 6]. This test is superior to clinical risk factors alone, accurately identifying 82% of cases [6]. The PROGNOSIS study demonstrated that a soluble fms-like tyrosine kinase 1 (sFlt-1) to PlGF ratio of 38 or lower effectively rules out the likelihood of developing preeclampsia within the next week, particularly in women under 37 weeks, with a 99.3% negative predictive value [7]. Despite the global momentum in seeking reliable predictors for pre-eclampsia, the biomarkers and tests used in these promising algorithms remain beyond reach in resource-limited settings, prompting a need for innovative approaches suitable for rural settings.

While the exact cause of preeclampsia remains unknown, a major etio-pathogenic player is the maladaptation of the spiral arteries during placenta formation [4]. This disorder seems to be a trophoblast-dependent process mediated by platelet dysfunction [8]. Furthermore, numerous studies have indicated that uncontrolled platelet activation and aggregation are also prevalent in cases of preeclampsia [9, 10]. Specifically, MPV and PDW serve as indicators of platelet activation. Larger platelets tend to be more reactive than smaller ones, likely due to an increased number and size of pseudopodia [11]. This phenomenon potentially leads to elevated PDW and MPV values. Growing evidence suggests that platelets play a role in the development of preeclampsia, and the condition may be preventable or mitigated through the use of antiplatelet agents, with low-dose aspirin being particularly notable [12]. In this vein, platelets have garnered attention in the context of pre-eclampsia, as evidenced by the consistently observed lower PC and higher MPV in affected individuals compared to normotensive pregnant women [13]. Several research findings are increasingly indicating that platelet indices hold promise as reliable predictors for pre-eclampsia [13–15]. The existing support for this notion primarily stems from case-control studies, highlighting the necessity for cohort studies to precisely establish the ideal timing and threshold values of platelet indices for clinical prediction of pre-eclampsia [15].

In Nigeria, most pre-eclampsia studies are cross-sectional or case-control, focusing on identifying associated factors and evaluating markers as predictors of severe disease [16–19]. Okwudire et al. conducted a prospective cohort study in southeast Nigeria, evaluating maternal uterine artery Doppler’s role in predicting preeclampsia [16]. Despite a small sample size (170 women), they found abnormal pulsatility indices had 53.8% sensitivity and 86.6% specificity [16]. Oluwole et al’s analytical cross-sectional study in southwest Nigeria linked low maternal placental growth factor levels (Adjusted OR 14.23) independently to preeclampsia [17]. Umezuluike et al. compared platelet parameters, noting significant differences between pre-eclamptic and normotensive women [18]. However, most of these studies lack predictive capacity due to design limitations, focusing on factors after preeclampsia onset with small sample sizes.Against this backdrop, our cohort study has centered on assessing the potential of MPV and platelet distribution width as predictors for early-onset pre-eclampsia in resource limited settings. These parameters are, readily accessible integral components of the standard full blood count tests.

## Materials and methods

This was a prospective cohort study conducted over eight months, March 2021 to October 2021, at the obstetrics department of the Lagos State University Teaching Hospital and General Hospital Ifako-Ijaiye, Lagos Nigeria. Consenting apparently healthy pregnant women who registered for antenatal care at the study centres, between gestational ages of 14weeks and 18weeks, were consecutively recruited into the study. Pregnant women with multiple pregnancy, diabetes mellitus, chronic hypertension, renal disease, known hematologic disease or anaemia with booking hematocrit < 30% were excluded from the study. In the study sites, women are routinely screened at their first visit for high and moderate risk factors of preeclampsia for possible commencement of oral Aspirin 75 mg daily. These women who qualified for oral Aspirin 75 mg daily for preeclampsia prevention were excluded from our study. This subgroup comprised individuals with either one major/high-risk factor (e.g., hypertensive disease in a previous pregnancy, chronic kidney disease, autoimmune conditions like systemic lupus erythematosus or antiphospholipid syndrome, type 1 or type 2 diabetes, or chronic hypertension) or two or more moderate risk factors (first pregnancy, age 40 years or older, pregnancy interval exceeding 10 years, BMI of 35 kg/m² or higher at the initial visit, family history of preeclampsia, or multiple pregnancies) for pre-eclampsia, following the NICE guideline [19].

The sample size was based on an anticipated 7.6% prevalence of pre-eclampsia in the study population [20]. The precision of the estimate needed to be within 5% points as assessed by the 95% confidence interval for the population prevalence—that is, a 95% confidence interval of 2.6–12.6%, assumed relative risk of 2, and power of 0.8. Using sample size calculator (EPITOOLS) [21] for a cohort study with the above values, the required total sample size was a minimum of 542. To allow for a potential 15% attrition rate, the calculated minimum required sample size was increased to 624. Given the lengthy 16–20week follow-up period, a strategic decision was made to intentionally oversample beyond the calculated sample size during recruitment. This precautionary measure ensured that, despite potential attrition or loss to follow-up, the study retained a sufficient number of participants to meet the minimum calculated sample size. At the end of the study 648 of the 670 women recruited completed the study.

Six hundred and forty-eight (648) of the eligible pregnant women recruited at 14-18weeks gestational age were followed-up to 34weeks gestation or when they developed pre-eclampsia, whichever came first. At recruitment, relevant bio-data and obstetric and medical history were obtained. The blood pressure of the women was measured according to standard using the Omron BP7100 blood pressure monitor (OMRON HEALTHCARE, INC. Lake Forest, IL 60,045 U.S.A.) and their venous blood samples were obtained into an EDTA bottle via venipuncture under aseptic condition according to standard. All blood samples were tested within less than 6 h of collection using hematology analyzer (Sysmex XN-1000 model (Sysmex Sverige, Sweden)) which performs blood cell count by direct current detection method, to determine the study participant’s PC, MPV and platelet distribution width.

The study participants had their routine antenatal care visits according to the antenatal care protocol of the study hospitals. They exited the study at 34weeks gestation or before 34weeks, if a diagnosis of pre-eclampsia was made. The study outcome of interest was development of pre-eclampsia. Preeclampsia in the study was diagnosed by the new onset of elevated blood pressure (≥ 140/90mmHg) and proteinuria (≥ 2+) after 20 weeks of gestation [22]. It was considered severe when blood pressure (≥ 160/110mmHg) and proteinuria increased substantially or symptoms of end-organ damage (including fetal growth restriction) occurred [22, 23].

Data obtained was analyzed using Statistical Package for Social Science (SPSS) version 20 (SPSS, Statistics for Windows, IBM Corp, Armonk, NY, USA). Percentages, mean and median of numerical variables were determined. Chi Square test was used to determine association of categorical variables and student t-test for continuous variables as appropriate. Mann Whitney U test was used to compare the median of two numerical variables. Correlation was evaluated using Pearson’s correlation. Sensitivity, specificity and negative predictive and positive predictive values were determined using 2 by 2 contingency tables and receiver operating characteristics (ROC) curves. The Youden’s test was used to determine cut-off of platelet indices with the best possible predictive values for pre-eclampsia [24]. For all statistical tests, a confidence level of 95% was used with *p* < 0.05 significance.

## Results

Out of 670 women recruited, 648 women completed the study and 22 women were lost to follow-up. Thirty-eight (38) of the 648 women studied developed early-onset pre-eclampsia, with a prevalence of 5.9%. Mild pre-eclampsia occurred in 26 women (4.0%) and severe pre-eclampsia was observed in 12 women (1.9%). Maternal age, educational status and gravidity were similar when women who developed pre-eclampsia were compared with women who did not develop pre-eclampsia (Table 1). Blood pressure measurements and platelet MPV and PDW at both recruitment (14-18weeks gestational age) and study exit (34weeks or delivery) were significantly lower in women who did not develop pre-eclampsia when compared with women who developed pre-eclampsia (Table 2). PC was lower in women who developed pre-eclampsia compared to women who did not develop pre-eclampsia (Table 2).

**Table 1:** Baseline characteristics of the study participants

Variables	Total Population *n* = 648(%)	Preeclampsia *n* = 38(%)	Normotensive *n* = 610(%)	χ^2^ /Fisher’s exact/ t- test	*P* value
**Age group (years)**					
< 20	1(0.2)	0(0.0)	1(0.2)	2.479^f^	0.882
20–29	357(55.1)	22(57.9)	335(54.9)		
30–39	289(44.6)	16(42.1)	273(44.8)		
≥ 40	1(0.2)	0(0.0)	1(0.2)		
Mean ± SD	28.9 ± 4.3	28.7 ± 4.1	28.9 ± 4.3	-0.327 ^t^	0.744
**Educational status**					
Primary	7(1.1)	0(0.0)	7(1.1)	0.770^f^	0.644
Secondary	35(5.4)	3(7.9)	32(5.2)		
Tertiary	606(93.5)	35(92.1)	571(93.6)		
**Gravidity**					
Primigravida	343(52.9)	19(50.0)	324(53.1)	0.139^x^	0.704
Multigravida	305(47.1)	19(50.0)	286(46.9)		
**Mean gestational age** *(weeks)*					
At Study Entry		15.18 ± 1.27	15.64 ± 1.52	1.83^t^	0.068
At Study Exit		32.7 ± 1.4	33.9 ± 0.3	-17.706^t^	< 0.001

SD-standard deviation, t-student’s t test applied, f-fischer’s exact applied, x^2^–chi-square applied

**Table 2:** Comparison of blood pressure and platelet characteristics among the women

Variables	Total *n* = 648 Median (IQR)	Study Cohorts		Mann Whitney U	*p*-value
		Women who developed pre-eclampsia *n* = 38 Median (IQR)	Women who did not develop pre-eclampsia *n* = 610 Median (IQR)		
**SBP** (mmHg)					
At recruitment	113(100–118)	122(119.8–130)	122(100–118)	1544.500	< 0.001
At study exit	125(122–128)	148.5(144–164)	124(122–128)	0.000	< 0.001
**DBP** (mmHg)					
At recruitment	64(60–80)	80(78–82)	64(60–66)	0.000	< 0.001
At study exit	65(62–68)	95.5(92–108)	64(62–67)	0.000	< 0.001
**MAP** (mmHg)					
At recruitment	79.7(76.7–82)	94(92–98.0)	79.3(76.3–81.3)	0.000	< 0.001
At study exit	84(83-86.7)	112.5(109.8-126.5)	84(82.7–86.7)	0.000	< 0.001
**Platelet count** (10^3^/µl)					
At recruitment	260(238–280)	190(177-217.5)	264(241–281)	741.500	< 0.001
At study exit	241(224–260)	165.5(157.5-181.3)	242(229–262)	71.00	< 0.001
**MPV** (fl.)					
At recruitment	8.2(7.4–8.7)	10.8(10.3–11.3)	8.1(7.4–8.5)	0.000	< 0.001
At study exit	8.7(8.4–9.5)	12.3(11.2–13.4)	8.7(8.4–9.3)	0.000	< 0.001
**PDW** (fl.)					
At recruitment	13.4(12.6–13.8)	24.8(22.6–31.7)	13.3(12.5–13.7)	0.000	< 0.001
At study exit	15.3(14.6–16.1)	26.9(24.9–33.7)	15.3(14.6–15.8)	0.000	< 0.001

*IQR-Interquartile range, SBP-Systolic blood pressure, DBP-Diastolic blood pressure, MAP-Mean arterial pressure, MPV-Mean platelet volume, PDW-Platelet Distribution Width, Recruitment = 14-18weeks gestation, Study exit = 34weeks or gestational age pre-eclampsia developed, fl-femtolitre*

**Fig. 1:**
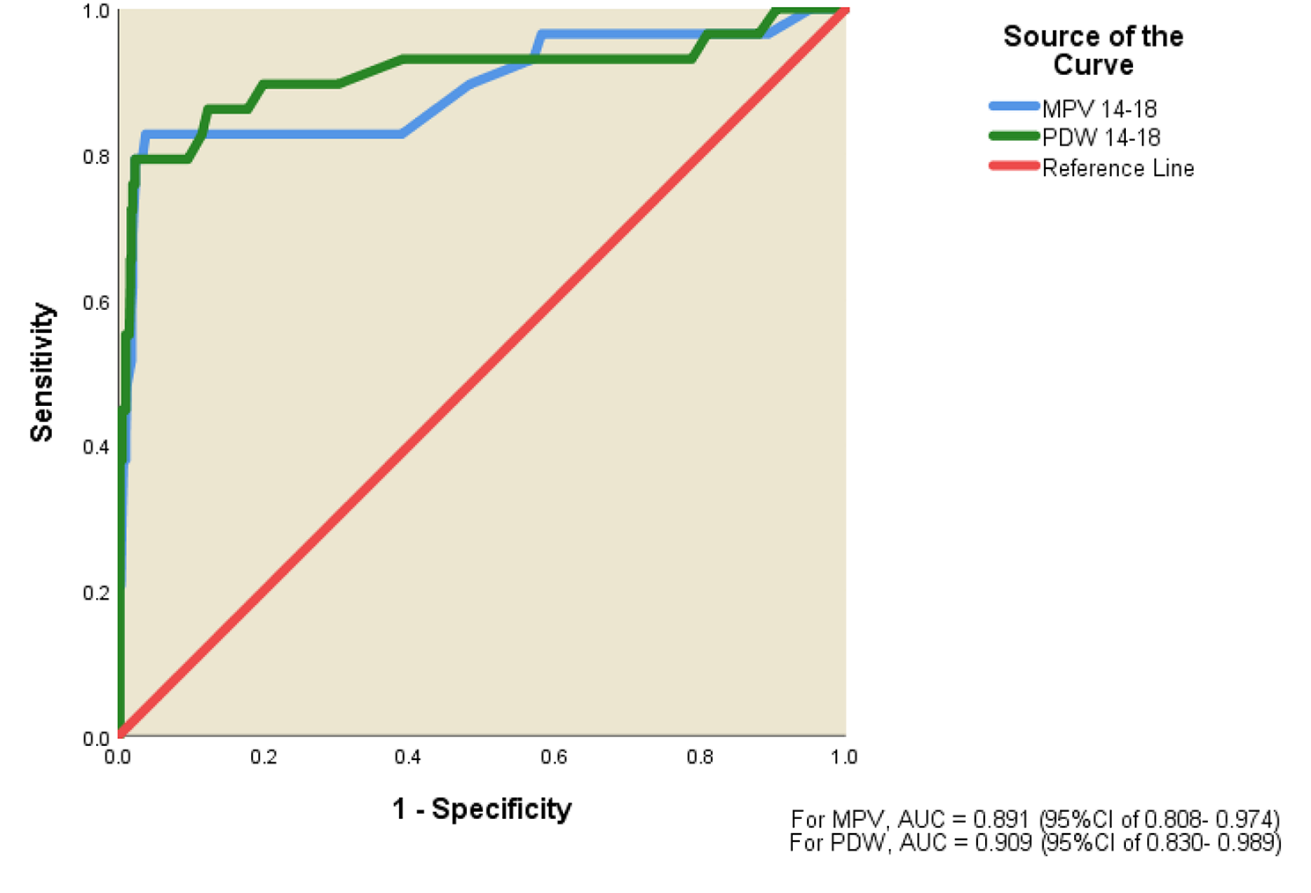
Receiver operative characteristic (ROC) curve showing MPV and PDW as predictors of early-onset pre-eclampsia

As regards evaluating platelet parameters at 14-18weeks as predictors of early-onset pre-eclampsia, the ROC curves, were above the reference line with the area under the curve (AUC) of 0.828, 0.891 and 0.909 showed that PC, MPV and PDW respectively were good predictor of early-onset pre-eclampsia in pregnant women (Fig. 1). The coordinate of ROC curve showed sensitivity and 1-specificity at various cut off value of platelet count, Youden index was determined using the Youden’s test. The Youden indices (62.2% for PC, 77.0% for PDW and 78.9% for MPV) were highest at PC, PDW and MPV cut off of 211.5, 21.3 and 9.4 respectively. These cut-off values had sensitivity of 96.6% and specificity of 65.6% for PC, sensitivity of 79.3% and specificity of 97.7% for PDW versus sensitivity of 82.8% and specificity of 96.1% for MPV (Table 3).

**Table 3:** Platelet characteristics at 14-18weeks gestation as predictor of early-onset pre-eclampsia

Parameters	Platelet count	PDW	MPV
Sensitivity (%)	96.6	79.3	82.8
Specificity (%)	65.6	97.7	96.1
Positive predictive value (%)	11.6	62.2	50.0
Negative predictive value (%)	99.8	99.0	99.2
Diagnostic accuracy (%)	67.0	96.9	95.5
Area under the curve	0.828	0.909	0.891
95% Confidence interval	0.717–0.940	0.830–0.989	0.808–0.974

*Cut-off values were determined using Youden’s test: Platelet count = 211.5 × 10* ^*3*^ */µl, PDW = 21.3 fl. and MPV = 9.4 fl*

*PDW– Platelet distribution width, MPV– Mean platelet volume*

**Fig. 2:**
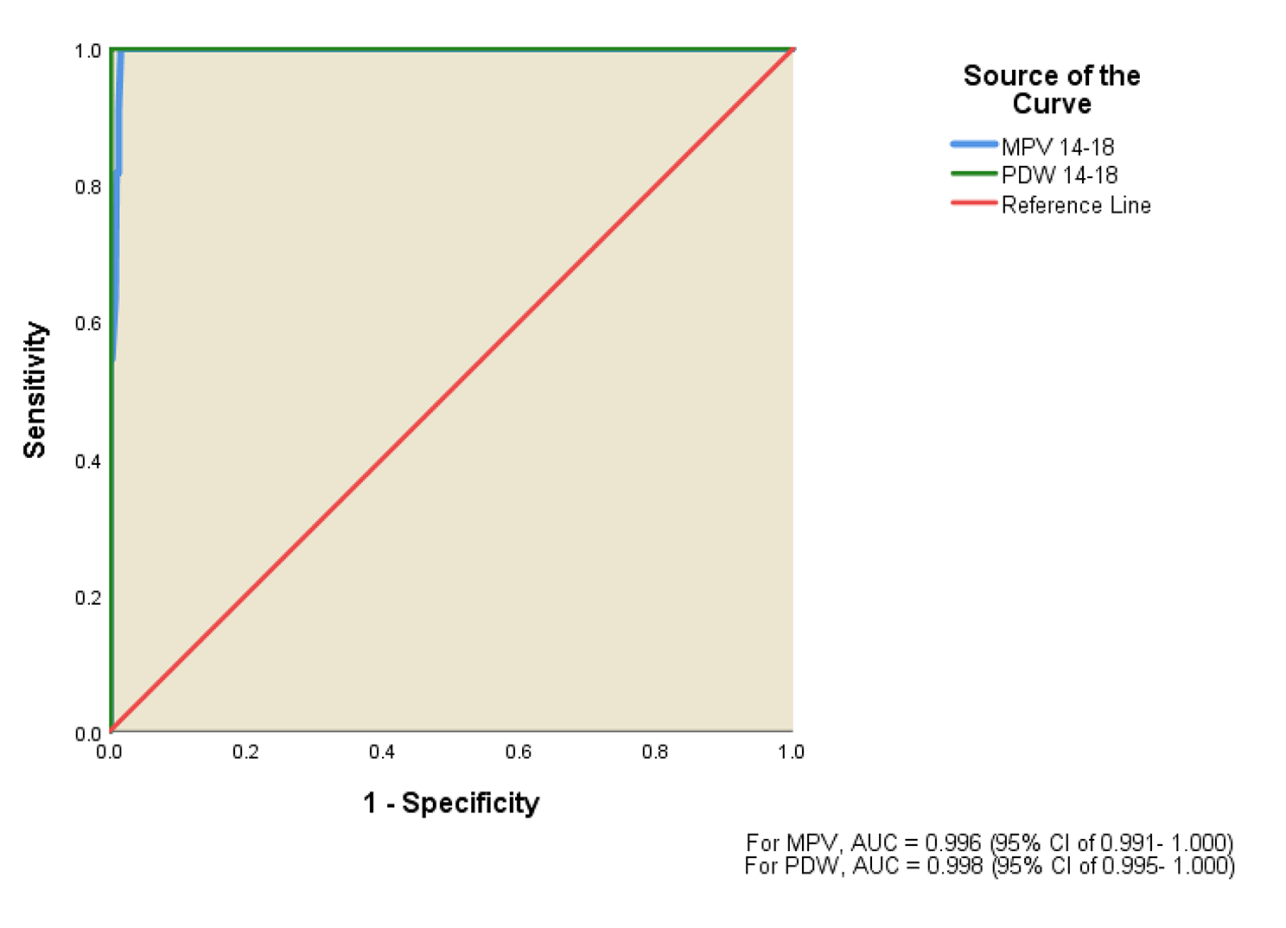
Receiver operative characteristic (ROC) curve showing MPV and PDW as predictors of severe early-onset pre-eclampsia

When the study platelet parameters were assessed as possible predictors of severe early-onset pre-eclampsia, ROC curves were above the reference line with AUC of 0.983, 0.996 and 0.998 which showed that PC, MPV and PDW respectively were good predictor of severe early onset preeclampsia in pregnant women (Fig. 2). The Youden indices (90.9% for PC, 98.4% for MPV and 99.8% for PDW) were highest at PC, MPV and PDW cut-off of 185, 10.7 and 28.3 respectively. These cut-off values had sensitivity of 100.0% and specificity of 90.9% for PC, sensitivity of 100.0% and specificity of 99.4% for MPV with sensitivity of 100.0% and specificity of 99.8% for PDW (Table 4).

**Table 4:** Platelet characteristics at 14-18weeks gestation as predictor of severe early-onset pre-eclampsia

Parameters	Platelet count	PDW	MPV
Sensitivity (%)	96.6	100.0	100.0
Specificity (%)	65.6	99.8	99.4
Positive predictive value (%)	11.6	91.7	73.3
Negative predictive value (%)	99.8	100.0	100.0
Diagnostic accuracy (%)	67.0	99.8	99.4
Area under the curve	0.983	0.998	0.996
95% Confidence interval	0.961- 1.000	0.995- 1.000	0.991- 1.000

*Cut-off values were determined using Youden’s test: Platelet count = 185 × 10* ^*3*^ */µl, PDW = 28.3 fl. and MPV = 10.7 fl*

*PDW– Platelet distribution width, MPV– Mean platelet volume*

## Discussion

Pre-eclampsia remains a major concern in maternal healthcare, and the findings of our prospective cohort study among Nigerian pregnant women provide valuable insights into the potential predictive role of platelet parameters, specifically MPV and PDW, in early-onset pre-eclampsia. The prevalence of early-onset pre-eclampsia in our cohort was 5.9%, with 4.0% classified as mild cases and 1.9% as severe cases. These results underscore the clinical relevance of identifying robust predictors for early-onset pre-eclampsia, given its association with increased perinatal and maternal morbidity.

Platelet activation in preeclampsia is believed to result from changes in the coagulation process between platelets and endothelial cells, triggered by widespread endothelial damage from defective trophoblastic invasion characteristic of the condition [25]. Activation of platelets can cause changes in size, count and distribution [26]. To assess platelet activation in early-onset preeclampsia, MPV and PDW (indicators of platelet size and distribution) were measured in healthy pregnant women at 14–16 weeks gestation and evaluated as possible predictors of early-onset preeclampsia in this study. We found out that platelet indices obtained at 14–18 weeks of gestational age exhibited notable distinctions between women who later developed early-onset pre-eclampsia and those who maintained normotension until 34 weeks. Specifically, there was a significant reduction in PC, accompanied by elevated MPV and PDW in individuals who subsequently developed pre-eclampsia, in contrast to the normotensive cohort. Our results align with those reported by Reddy et al. [27] who observed that MPV and PDW were significantly higher in the preeclampsia group; Bellos et al. [27], who conducted a meta-analysis involving 50 studies encompassing 14,614 women; and Woldeamanuel et al., who reviewed 56 studies comprising 4892 preeclamptic and 9947 normotensive pregnant women [28]. In contrast, Temur et al. observed a significant difference solely in MPV values [29]. Conversely, Lin et al’s study in China reported that no individual platelet parameter during the early stages of pregnancy demonstrated significant difference between pre-eclamptics and non-pre-eclamptics [29]. However, Lin et al. noted that MPV from 16 to 19 weeks of gestation, the difference between pre-eclampsia and non-preeclampsia group was significant and the differences between both groups became more obvious with advancing gestational age. The differences between our findings and those of Temur and Lin may be attributed to the retrospective, case-control design of Temur’s study and the distinct Asian population in Lin et al’s investigation.

The evaluation of platelet parameters as predictors of early-onset pre-eclampsia demonstrated promising results. The ROC curves displayed good predictive capabilities, with AUC values of 0.828, 0.891, and 0.909 for PC, MPV, and PDW, respectively. The Youden indices were highest at specific cut-offs (PC - 211.5 × 10^3^/µl, PDW − 21.3 fl. and MPV − 9.4 fl.), indicating the potential clinical utility of these platelet parameters as predictive markers. Notably, the sensitivity and specificity values (PC − 96.6% and 65.6%, PDW − 79.3% and 97.7% and MPV − 82.8% and 96.1% respectively) further support the validity of these thresholds, particularly in the context of early-onset pre-eclampsia. In Temur et al’s work, the optimal cut-off value for MPV was determined to be 9.15 fl., offering a sensitivity of 58.7% and specificity of 61.7% for predicting preeclampsia [14]. In Lin et al’s study, the prediction of preterm pre-eclampsia yielded AUC values of 0.616, 0.612, and 0.552, with corresponding sensitivity rates of 37.8%, 45.9%, and 83.8%, and specificity rates of 82.9%, 74.3%, and 28.1% for PC, MPV, and PDW, respectively [29].

For severe early-onset pre-eclampsia, the predictive performance of platelet parameters was even more striking, with AUC values of 0.983, 0.996, and 0.998 for PC, MPV, and PDW, respectively. The Youden indices at specific cut-offs demonstrated exceptional sensitivity and specificity, highlighting the potential for these platelet parameters to serve as reliable predictors of severe early-onset pre-eclampsia. It was observed that PC < 185,000, MPV > 10.7 fl. and PDW > 28.3 fl., had sensitivity of 100.0% and specificity of 90.9% for PC, sensitivity of 100.0% and specificity of 99.4% for MPV with sensitivity of 100.0% and specificity of 99.8% for PDW. Reddy et al. found 80% sensitivity, 75% specificity and AUC of 0.78 in predicting severe pre-eclampsia with a cut-off MPV > 10.95fl [27]. They also observed that PDW > 17.75 fl. predicted severe pre-eclampsia with 66% sensitivity, 62% specificity and AUC of 0.742 [27]. The differences in our findings compared to those of Reddy et al., Temur et al. and Lin et al. could be attributed to several factors, including variations in the study populations and methodologies. Firstly, demographic and genetic differences among Nigerian women may contribute to distinct platelet parameter profiles compared to the populations studied by Temur et al. and Lin et al. Additionally, methodological variations, such as the specific diagnostic criteria for pre-eclampsia (our study only evaluated early-onset pre-eclampsia) and differences in gestational ages at which platelet parameters were measured could also play a role. These multifaceted factors emphasize the need for further research and validation in diverse populations to establish the generalizability and reliability of platelet parameters as predictive markers for pre-eclampsia.

MPV is a measure of the average size of platelets, and an increase in MPV is commonly associated with platelet activation [26]. PDW reflects the variation in platelet size, and an elevated PDW suggests increased heterogeneity in platelet size, which is also indicative of platelet activation [26]. The observed increase in MPV and PDW associated with preeclampsia in our study are likely manifestations of the activated state of platelets in response to the endothelial damage and altered coagulation processes associated with this pregnancy-related condition [25]. However, in this study both MPV and PDW were measured early in pregnancy before the development of preeclampsia. This suggests that changes in MPV and PDW precedes the clinical manifestation of preeclampsia, buttressing the need to further evaluate them as predictive markers. Dundar et al. had also previously reported that increase in MPV may precede preeclampsia symptoms by approximately 4.6 weeks [30].

Our study has limitations, primarily stemming from potential selection bias, as participants were enrolled between gestational weeks 14 to 18 due to the limited popularity of early antenatal care registration in Nigeria. This hinders the broad applicability of our findings. Additionally, the identification of only a few cases of severe early-onset preeclampsia in our study warrants cautious interpretation of the high predictive values observed with this condition. Furthermore, the overrepresentation of participants with tertiary-level education raises concerns about the generalizability of our results to the broader national population. The major strength of our study lies in its robust prospective design.

In conclusion, the evaluation of MPV and PDW between 14 and 18 weeks of gestation of pregnancy appears to be a reliable predictor of severe early-onset pre-eclampsia among black Nigerian women. The findings in our study population signify a significant stride toward developing accessible and effective predictive tools for pre-eclampsia in resource-limited settings. By leveraging routine full blood count tests, which are more readily available, our study contributes to the ongoing global efforts to identify markers that can aid in early risk stratification, allowing for timely interventions and improved maternal and fetal outcomes. Further validation through larger, diverse cohorts and longitudinal studies will strengthen the foundation laid by our research, paving the way for the integration of platelet parameters into routine antenatal care practices.

## Data Availability

Study data is available and can be obtained on reasonable request from the corresponding author.

## Abbreviations

AUC: Area under the curve
DBP: Diastolic blood pressure
EDTA: Ethylenediamine tetraacetic acid
LASUTH: Lagos state university teaching hospital
MAP: Mean arterial pressure
MPV: Mean Platelet Volume
NICE: National Institute for Health and Care Excellence
PDW: Platelet Distribution Width
PE: Pre-eclampsia
PlGF: Placenta growth factor
ROC: Receiver operating characteristics
SBP: Systolic blood pressure
SD: Standard deviation
sFlt-1: Soluble fms-like tyrosine kinase 1
SPSS: Statistical package for social sciences
